# Early detection and management of radiation maculopathy after plaque brachytherapy for uveal melanoma: a multimodal imaging narrative review

**DOI:** 10.3389/fonc.2026.1926816

**Published:** 2026-10-29

**Authors:** Jesus Hernan Gonzalez-Cortes, Jesus Emiliano Gonzalez-Cantu, Anibal Martin Folgar, David Pelayes, Jesus Mohamed-Hamsho

**Affiliations:** 1 Department of Ophthalmology, University Hospital and Faculty of Medicine, Autonomous University of Nuevo Leon, Monterrey, Mexico; 2 Department of Ophthalmology, Universidad Maimonides, Buenos Aires, Argentina

**Keywords:** anti-VEGF, laser speckle flowgraphy, optical coherence tomography angiography, plaque brachytherapy, radiation maculopathy, radiation retinopathy, retinal ischemia, uveal melanoma

## Abstract

**Background:**

Radiation retinopathy is a dose- and time-dependent neurovascular complication of ocular radiotherapy. Radiation maculopathy denotes macular involvement and may present with early retinal thickening or edema, progressive nonperfusion, or late atrophy. Its biology is not a single linear sequence: endothelial, inflammatory, permeability, ischemic, and neuronal changes overlap.

**Objective:**

To critically review terminology, classification, plaque-related dosimetry, multimodal imaging biomarkers, and preventive and therapeutic evidence for radiation maculopathy after plaque brachytherapy for uveal melanoma.

**Methods:**

PubMed/MEDLINE was searched from January 2000 through 24 September 2026 using combinations of uveal melanoma, radiation retinopathy, radiation maculopathy, plaque brachytherapy, iodine-125, ruthenium-106, palladium-103, cesium-131, OCT, OCT angiography, fluorescein angiography, laser speckle flowgraphy, anti-VEGF, bevacizumab, ranibizumab, aflibercept, corticosteroid, and laser. Seminal pre-2000 studies and additional records identified from reference lists were retained when they defined classification, natural history, or treatment. Original clinical studies were prioritized for efficacy and imaging claims; reviews were used for context and evidence synthesis.

**Results:**

Structural OCT can reveal macular edema before ophthalmoscopic radiation retinopathy, but thickness changes also reflect neuronal swelling and late atrophy. OCTA detects capillary-density loss and foveal avascular zone change, yet measurements depend on signal quality and segmentation and are especially vulnerable to cystoid edema. Fluorescein angiography remains important for leakage and wide-field ischemic burden, while laser speckle flowgraphy is an investigational perfusion measure. Anti-VEGF therapy is the best-supported treatment for established radiation-related macular edema and generally requires sustained dosing. Prophylactic anti-VEGF cohorts show lower rates of edema and some radiation complications, but long-term visual superiority is not consistently demonstrated and randomized plaque-specific evidence is lacking. Corticosteroids and laser have selective roles.

**Conclusions:**

Surveillance should be multimodal and individualized according to tumor location, patient-specific dosimetry, visual potential, and serial structural and perfusion findings. OCTA complements rather than replaces structural OCT and angiography. No validated OCTA threshold currently justifies treatment in an otherwise unaffected eye; prospective studies must define reproducible biomarkers and treatment triggers.

## 1 Introduction

Plaque brachytherapy is an established globe-preserving treatment for selected uveal melanomas. The Collaborative Ocular Melanoma Study demonstrated survival comparable to enucleation for medium choroidal melanoma treated with I-125 plaque therapy, but visual loss remained frequent because tumor location and irradiation of the macula, optic nerve, lens, and ciliary body determine functional outcome (1, 2). Contemporary pooled evidence confirms high local-control rates across plaque systems, while also showing that toxicity estimates vary with isotope, case selection, prescription, plaque geometry, and duration of follow-up (3).

Radiation retinopathy refers to radiation-induced injury involving the retinal neurovascular unit beyond the macula. Radiation maculopathy is the macular phenotype and may include leakage, edema, nonperfusion, hemorrhage, exudation, photoreceptor disruption, and atrophy. The terms overlap in the literature but are not fully interchangeable. This distinction matters because a review restricted to macular edema can miss early or peripheral vascular changes, peripapillary injury, and neuronal abnormalities relevant to later visual loss.

The central clinical problem is therefore not simply to detect edema earlier. It is to identify which structural and perfusion abnormalities are reproducible, which are linked to radiation dose and visual prognosis, and which justify intervention. This review re-examines these questions using original imaging and treatment studies and explicitly separates plaque brachytherapy from proton and other external-beam modalities. The classification frameworks, plaque isotope characteristics, selected imaging studies, and therapeutic evidence are summarized in **Tables 1**–**4**, while the temporal disease model and representative multimodal findings are illustrated in **Figures 1**–**3**.

**Table 1:** Comparison of classification and imaging frameworks for radiation maculopathy.

Framework	Modality	Primary dimension	Strength	Important limitation
Horgan et al. (4)	Structural OCT	Location and cystoid character of macular edema	Sensitive to early edema; clinically accessible	Does not quantify panretinal ischemia, neuronal injury, or late atrophy
McCannel et al. (5)	UWF fluorescein angiography	Panfundus vascular severity	Captures leakage and nonperfusion beyond the macula	Invasive; leakage can obscure capillary detail
OCTA metrics	OCTA	Vessel density, FAZ, plexus-specific loss	Noninvasive and depth resolved	Device, signal, segmentation, projection, and edema artifacts; no validated treatment cutoffs

FAZ, foveal avascular zone; OCT, optical coherence tomography; OCTA, optical coherence tomography angiography; UWF, ultra-wide-field.

**Table 2:** Physical and clinical characteristics of commonly used plaque brachytherapy isotopes.

Isotope	Emission and practical profile	Typical clinical consideration	Evidence caveat
I-125	Low-energy photons; broad North American experience	Small-to-medium tumors; COMS-based historical framework	Dose reporting and plaque heterogeneity methods vary
Ru-106	Beta emission with steep distal falloff	Generally favored for thinner tumors	Selection by tumor thickness limits direct comparisons
Pd-103	Lower photon energy than I-125	Potential dosimetric sparing in selected geometries	Predominantly single-center nonrandomized evidence
Cs-131	Short half-life and high initial dose rate	Shorter treatment duration in some systems	Limited ocular outcome and maculopathy data

COMS, Collaborative Ocular Melanoma Study; Cs-131, cesium-131; I-125, iodine-125; Pd-103, palladium-103; Ru-106, ruthenium-106.

**Table 3:** Selected multimodal imaging studies in radiation retinopathy and radiation maculopathy.

Study	Radiation	Design and sample	Main imaging finding	Key limitation
Say et al., 2016 (20)	Plaque	Cross-sectional; eyes without clinical RM	Parafoveal capillary-density reduction on OCTA	Cross-sectional and device specific
Cennamo et al., 2018 (22)	Ru-106 plaque	Prospective; 39 eyes	Vessel-density reduction one year after treatment	No untreated irradiated comparator
Skalet et al., 2020 (23)	I-125 plaque	Prospective longitudinal; 21 eyes	Macular/peripapillary density loss and FAZ enlargement; dose association	Small cohort and annual intervals
Parrozzani et al., 2022 (17)	I-125 plaque	Prospective; 30 eyes	Hyperreflective foci preceded macular edema	Single-center biomarker study
Tamplin et al., 2022 (19)	I-125 plaque	Cross-sectional; 25 patients	Reduced retinal and choroidal flow on LSFG	Relative flow metric; cross-sectional
Tamplin et al., 2024 (18)	I-125 plaque	Cross-sectional multimodal cohort	Early vascular and neuronal abnormalities may be dissociated	Small heterogeneous time points

FAZ, foveal avascular zone; I-125, iodine-125; LSFG, laser speckle flowgraphy; OCTA, optical coherence tomography angiography; RM, radiation maculopathy; Ru-106, ruthenium-106.

**Table 4:** Selected therapeutic studies for radiation retinopathy and radiation maculopathy.

Study	Modality and design	N	Intervention	Accurate interpretation
Finger & Chin 2007 (25)	Plaque; prospective case series	6	Bevacizumab	Early proof of concept; very small series
Shah et al., 2014 (27)	I-125 plaque; prospective case series	15	Monthly bevacizumab	Anatomic improvement with repeated dosing; uncontrolled
Murray et al., 2019/2021 (28, 29)	Mixed ocular radiation; randomized year 1 and extension	40 enrolled; 39 completed year 1; 30 entered year 2	Aflibercept fixed vs treat-and-adjust, then extension	Frequent ongoing dosing required; attrition relevant to year-2 estimates
Fallico et al., 2019 (30)	Ru-106 plaque; prospective study	9	Aflibercept	Improved thickness and visual outcomes; small cohort
RRR Study 2020/2022 (31, 32)	Radiation-related edema; randomized phase IIb	40 eyes	Monthly vs PRN ranibizumab with/without targeted laser	Monthly dosing produced the strongest first-year result
Shields et al., 2020 (34)	I-125 plaque; retrospective cohort vs 117 historical controls	1,131 treated	Bevacizumab every 4 months for 2 years	Less edema/RM at several time points; poor vision at 4 years 61% vs 61%, P = .11
Horgan et al., 2009 (36)	I-125 plaque; randomized controlled trial	163	Periocular triamcinolone	Reduced OCT edema through 18 months; steroid risks and older treatment context

I-125, iodine-125; OCT, optical coherence tomography; PRN, pro re nata (as needed); RM, radiation maculopathy; RRR, Radiotherapy-Related Retinopathy; Ru-106, ruthenium-106; VEGF, vascular endothelial growth factor.

**Figure 1:**
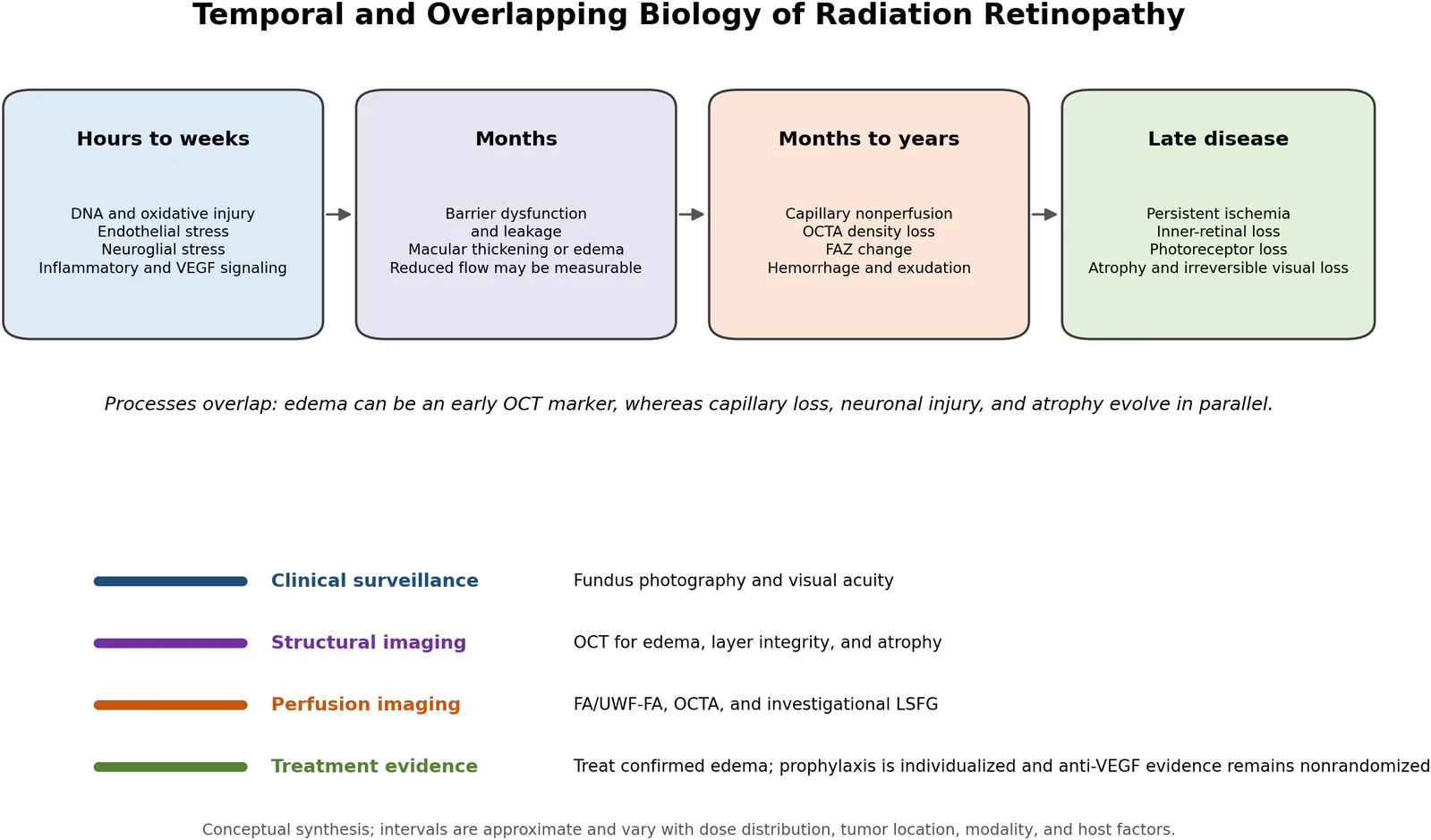
Temporal and overlapping biology of radiation retinopathy. Radiation-induced endothelial, permeability, inflammatory, and neuroglial changes overlap. Macular thickening or edema can be an early OCT manifestation; capillary nonperfusion, neuronal loss, and atrophy progress variably with dose, location, and time. The schematic is a conceptual synthesis rather than a validated staging timeline (4, 15–19).

**Figure 2:**
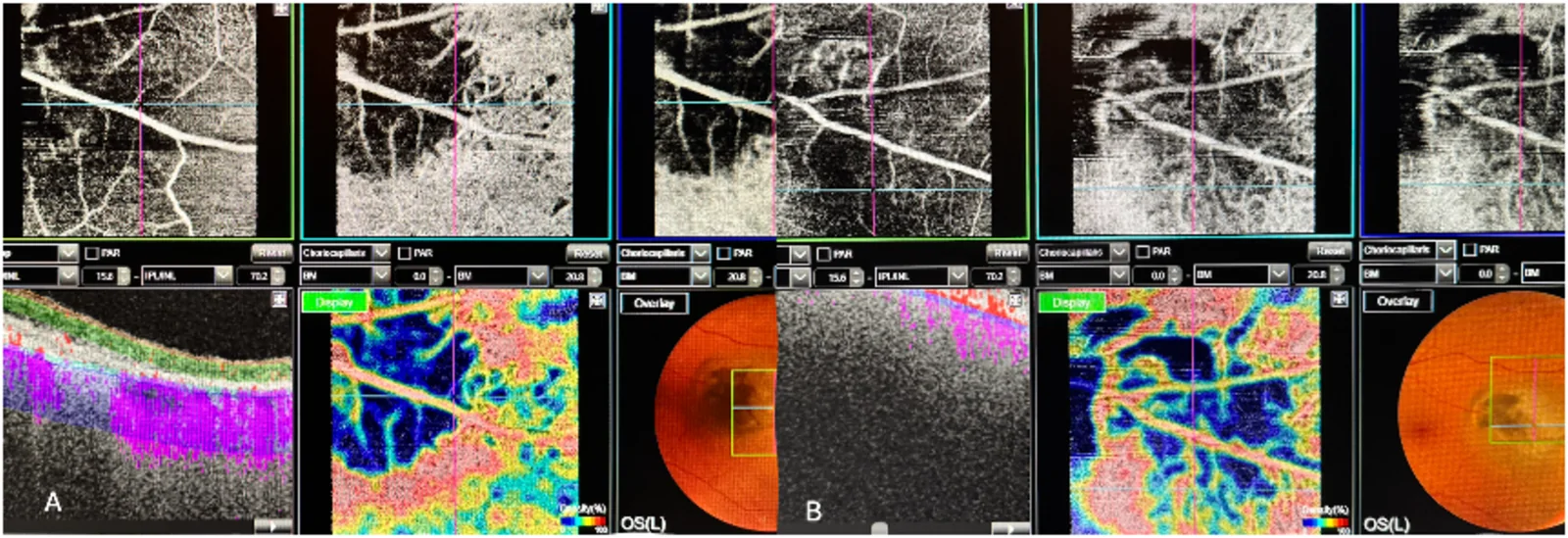
Representative OCTA examinations illustrating retinal microvascular abnormalities after plaque brachytherapy. **(A, B)** show areas of reduced capillary density and nonperfusion on en face OCTA, with corresponding B-scan flow overlays and density maps. The exact interval between examinations and the treatment history were not available for this narrative review. Interpretation should include signal-strength and segmentation review and correlation with structural OCT.

**Figure 3:**
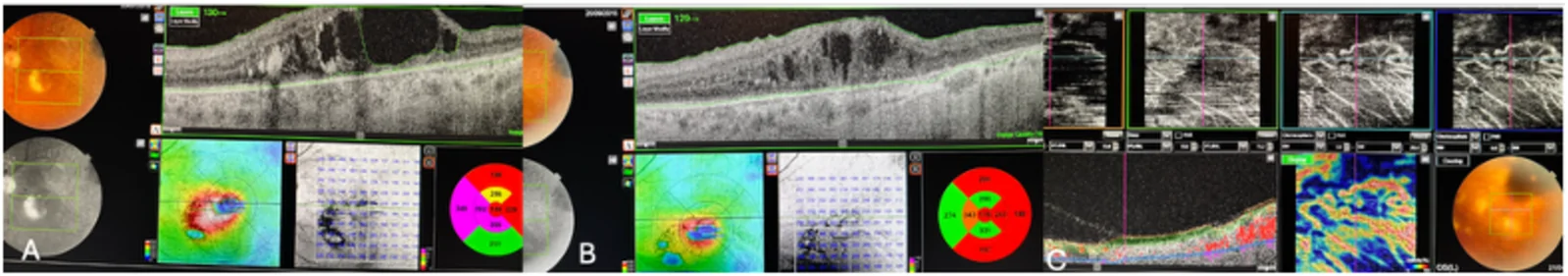
Representative multimodal images illustrating different stages of radiation maculopathy. **(A, B)** demonstrate cystoid macular edema on structural OCT with corresponding thickness maps; **(C)** demonstrates chronic retinal thinning and atrophy with OCTA evidence of capillary rarefaction and nonperfusion. The exact intervals between images and the treatment history were not available for this narrative review. Fluid resolution does not establish reperfusion or recovery of neuronal tissue.

## 2 Scope and literature search

This narrative review was designed as a clinically oriented critical synthesis rather than a systematic review or meta-analysis. PubMed/MEDLINE was searched from January 2000 through 24 September 2026. The search combined disease terms (radiation retinopathy, radiation maculopathy, radiation-induced macular edema), tumor and treatment terms (uveal melanoma, choroidal melanoma, plaque brachytherapy, I-125, Ru-106, Pd-103, Cs-131, proton beam), imaging terms (fundus photography, fluorescein angiography, ultra-wide-field angiography, OCT, OCTA, vessel density, foveal avascular zone, laser speckle flowgraphy), and treatment terms (anti-VEGF, bevacizumab, ranibizumab, aflibercept, corticosteroid, triamcinolone, dexamethasone, laser).

Eligible publications included original prospective or retrospective clinical studies, randomized or comparative trials, dosimetry studies, classification studies, systematic reviews, and major consensus reports. Seminal older publications were included when they established natural history, classification, or treatment. Original studies were preferentially cited for numerical results and therapeutic claims. Studies of proton therapy were retained only when they addressed a relevant biological, imaging, or therapeutic question and are identified as such rather than pooled conceptually with plaque therapy. Because study designs, radiation modalities, outcome definitions, and treatment schedules were heterogeneous, no quantitative pooling was attempted.

## 3 Terminology and classification

Radiation injury involves endothelial cells, pericytes, glia, neurons, and the retinal pigment epithelium. Accordingly, the phrase microvascular compromise should be used descriptively only when a measured vascular abnormality is specified, such as reduced capillary density, nonperfusion, leakage, or impaired flow. It should not be treated as a mechanism distinct from endothelial dysfunction.

Horgan et al. proposed an OCT-based classification of radiation macular edema: grade 1, extrafoveal noncystoid edema; grade 2, extrafoveal cystoid edema; grade 3, foveal noncystoid edema; grade 4, mild-to-moderate foveal cystoid edema; and grade 5, severe foveal cystoid edema (4). This system emphasizes edema location and morphology, but it does not capture peripheral ischemia, neuronal loss, or late atrophy.

McCannel et al. developed an ultra-wide-field fluorescein angiographic grading scheme for panfundus radiation retinopathy after I-125 brachytherapy (5). It complements the macula-centered OCT classification by representing the distribution and severity of vascular abnormalities. Neither system should be interpreted as a universal treatment algorithm, and cross-device OCTA thresholds have not been validated.

## 4 Plaque isotopes dosimetry and therapeutic ratio

Tumor control remains the first requirement of treatment planning. The therapeutic ratio is optimized by delivering an adequate dose to the entire tumor and margin while limiting dose to uninvolved structures where technically possible. This is not equivalent to empirically lowering the tumor prescription. Relevant variables include plaque design and size, seed loading, radionuclide energy, dose rate, duration, tumor thickness, plaque-tumor apposition, and the dose-volume distribution to the fovea, optic disc, lens, and ciliary body (6–8).

I-125 is a low-energy photon emitter with extensive clinical experience and was used in COMS. Ru-106 is a beta emitter with steep dose falloff and is most applicable to thinner tumors. Pd-103 has lower photon energy than I-125 and can reduce dose to some opposite-wall structures in selected geometries, although clinical comparisons are nonrandomized. Cs-131 has a shorter half-life and higher initial dose rate; clinical experience is comparatively limited (9–11). Physical characteristics alone do not determine macular toxicity, because tumor location, plaque placement, prescription, and patient-specific geometry are dominant confounders.

## 5 Pathobiology and temporal phenotype

Radiation injury should not be presented as a single obligatory sequence. Ionizing radiation causes DNA and oxidative injury and affects endothelial, pericyte, inflammatory, glial, and neuronal pathways. Increased permeability and macular thickening may appear early, sometimes before visible fundus changes. In parallel, progressive capillary closure, impaired perfusion, and neuronal degeneration evolve at rates determined by dose distribution and host susceptibility (12–16).

VEGF signaling is best considered part of an early and sustained stress response rather than a late event that begins only after established ischemia. Clinical prophylaxis studies are biologically compatible with early signaling, but they do not by themselves prove the precise timing or magnitude of intraocular VEGF after plaque removal. Later disease may include persistent edema, extensive ischemia, photoreceptor and inner-retinal loss, and atrophy; fluid resolution does not imply restoration of perfusion or neural tissue.

## 6 Natural history and risk factors

Reported incidence varies because studies use different definitions, imaging schedules, radiation modalities, tumor distributions, and follow-up. Consistent risk factors include shorter tumor-to-fovea and tumor-to-disc distances, larger tumor dimensions, higher macular or optic-disc dose, and systemic vascular comorbidity such as diabetes. Visual prognosis is additionally influenced by baseline acuity, direct tumor involvement of the macula or disc, radiation optic neuropathy, and late retinal atrophy (3, 12–14).

Risk should therefore be estimated from the actual treatment plan rather than inferred from isotope alone. Baseline documentation should include visual acuity, tumor location, structural OCT, and the planned dose to the fovea and optic disc when available. The same variables are necessary for interpreting longitudinal imaging.

## 7 Multimodal imaging

### 7.1 Fundus photography and fluorescein angiography

Color photography documents hemorrhage, exudation, cotton-wool spots, vascular attenuation, neovascularization, and atrophy but is insensitive to subclinical edema and subtle perfusion change. Fluorescein angiography remains valuable because it demonstrates leakage and nonperfusion. Ultra-wide-field angiography extends assessment beyond the macula and supports panfundus grading, although it remains invasive and leakage may obscure microvascular detail (5, 16).

### 7.2 Structural optical coherence tomography

Structural OCT is central to surveillance because macular thickening can precede ophthalmoscopic retinopathy and visual symptoms. Early findings include noncystoid thickening, intraretinal cysts, and occasionally subretinal fluid; later findings include disorganization, photoreceptor damage, inner-retinal thinning, and atrophy (4, 15, 20).

Retinal thickness is not a purely vascular endpoint. Increased thickness can reflect leakage, cyst formation, or neuronal and glial swelling, while reduced thickness can reflect neurodegeneration and tissue loss. Hyperreflective retinal foci have been reported before radiation macular edema and may represent an inflammatory imaging biomarker, but external validation and treatment implications remain limited (17, 18).

### 7.3 Optical coherence tomography angiography

OCTA provides depth-resolved, dye-free visualization of retinal capillary networks. Studies after plaque or proton therapy have reported reduced vessel density, deep-plexus abnormalities, FAZ enlargement, and peripapillary capillary loss. Prospective longitudinal I-125 data show that macular and peripapillary density changes can emerge by 12 months and correlate with dose and visual function (20–24).

These measurements are not infallible. Cystoid spaces and retinal deformation can impair automated segmentation; low signal, motion, projection artifacts, scan size, device software, and thresholding alter vessel-density and FAZ estimates. Apparent FAZ enlargement should be confirmed on the en face image and B-scan segmentation and interpreted with structural OCT. Anti-VEGF can improve thickness without restoring vessel density or FAZ area, illustrating that anatomic drying and reperfusion are different outcomes (24).

### 7.4 Laser speckle flowgraphy and emerging functional imaging

Laser speckle flowgraphy measures relative blood-flow indices rather than capillary anatomy. In a cross-sectional cohort of 25 patients after I-125 plaque therapy, retinal and choroidal flow were lower in irradiated than fellow eyes, including five of six patients imaged within six months. This technique is promising for early functional assessment, but normative ranges, longitudinal reproducibility, and treatment thresholds require validation (19).

Recent dose-linked OCT, OCTA, and perimetry analyses also demonstrate that microvascular and neuronal abnormalities may occur independently early after treatment and converge later. This supports a neurovascular rather than exclusively vascular disease model (18).

## 8 Treatment

### 8.1 Anti-VEGF for established macular edema

Anti-VEGF therapy has the strongest clinical evidence for established radiation-related macular edema, although all use remains off label. Finger and Chin reported an initial six-patient case series of intravitreal bevacizumab for radiation retinopathy (25). Longer-term cohorts indicate that repeated bevacizumab or ranibizumab can preserve vision in many treated eyes, but recurrence is common when intervals are extended or treatment is discontinued (26, 27).

The randomized ARM study enrolled 40 patients and compared fixed six-week aflibercept with a treat-and-adjust strategy; 39 completed year 1 and 30 entered the year-2 extension. Both regimens required frequent ongoing treatment, with maintained mean acuity and reduced thickness among continuing participants (28, 29). After Ru-106 plaque therapy, aflibercept has also shown anatomical and functional benefit in prospective clinical data (30).

The RRR trial randomized 40 eyes to monthly ranibizumab, monthly ranibizumab plus targeted retinal photocoagulation, or loading injections followed by as-needed ranibizumab plus photocoagulation. Monthly therapy produced the largest first-year visual gain; subsequent treat-and-extend treatment maintained benefit less consistently than continuous monthly dosing (31, 32). These studies support sustained individualized therapy rather than assuming durable control after a short loading phase.

### 8.2 Prophylactic anti-VEGF

In plaque-treated eyes, scheduled bevacizumab at plaque removal and every four months for two years was associated with lower rates of OCT edema and some radiation complications than historical controls (33, 34). However, in the four-year analysis, Kaplan-Meier estimated poor vision of 20/200 or worse was 61% in both groups and was not significantly different. Thus, these data support reduced intermediate anatomic morbidity but do not establish long-term visual superiority. The 2020 age analysis represents an overlapping Wills Eye cohort and should not be counted as an independent efficacy study.

Prophylactic ranibizumab after proton irradiation showed encouraging outcomes in a small pilot study, but proton and plaque therapy differ in dose distribution and should not be directly pooled (35). No validated OCTA threshold currently identifies an asymptomatic plaque-treated eye that must receive prophylaxis. Decisions should consider tumor location, foveal dose, visual potential, systemic risk, and patient tolerance for repeated injections.

### 8.3 Corticosteroids

Periocular triamcinolone administered at plaque treatment and at four and eight months reduced OCT-detected edema through 18 months in a randomized trial, but steroid-related ocular risk and modern anti-VEGF practice affect applicability (36). Intravitreal triamcinolone and dexamethasone implants can reduce edema in selected eyes, including eyes with incomplete anti-VEGF response, but cataract and intraocular-pressure elevation require consideration (37–39).

### 8.4 Laser and other approaches

Focal or sector laser and panretinal photocoagulation have historical roles for focal leakage, ischemic retina, or neovascularization. Anti-VEGF was superior to laser during active dosing in the RadiRet trial, but the advantage diminished after injections stopped, underscoring the chronic nature of the disease (40, 41). Laser is therefore not a substitute for pharmacologic treatment of center-involving edema, although it remains appropriate for selected peripheral ischemic or proliferative phenotypes.

Evidence for switching anti-VEGF agents, combined steroid therapy, or newer agents is derived mainly from small series. These options may be reasonable in refractory disease, but comparative effectiveness and safety are not established (42–44).

## 9 Multimodal surveillance framework

Because validated OCTA risk thresholds are not currently available, a clinically oriented framework is to obtain baseline structural OCT and, when available, OCTA before or soon after treatment; document patient-specific foveal and disc dose; repeat structural OCT at clinically appropriate intervals; and use FA/UWF-FA or OCTA when edema, unexplained visual change, or suspected ischemia requires clarification.

Treatment should be triggered by clinically meaningful edema, visual change, neovascular complications, or an individualized prophylactic decision based on established clinical and dosimetric risk, not by an isolated unvalidated OCTA value. Serial change within the same device and protocol is more informative than a single measurement. Segmentation must be checked manually whenever edema or poor signal is present.

## 10 Limitations and future directions

The literature is limited by retrospective designs, small imaging cohorts, historical controls, inconsistent definitions, heterogeneous radiation modalities, and variable treatment schedules. Comparisons across isotopes are confounded by tumor thickness and location. Treatment studies frequently use thickness rather than perfusion or patient-reported function as the main outcome. OCTA metrics are device dependent, and clinically meaningful change thresholds are undefined.

Future prospective studies should combine patient-specific dosimetry with standardized OCT, OCTA, angiographic, and functional outcomes; prespecify segmentation-quality rules; distinguish plaque from proton therapy; and report edema, perfusion, neuronal integrity, visual acuity, and quality of life separately. Randomized plaque-specific trials are needed to identify who benefits from prophylaxis, when it should begin, and how long it should continue.

## 11 Conclusions

Radiation maculopathy is a heterogeneous macular manifestation of radiation retinopathy involving overlapping vascular, permeability, inflammatory, and neuronal processes. Edema can be an early OCT marker, but late visual loss also reflects ischemia and neuroretinal atrophy. OCTA adds noninvasive plexus-specific information but is vulnerable to segmentation and signal artifacts and currently lacks validated treatment thresholds. Anti-VEGF therapy is the best-supported treatment for established edema and usually requires sustained administration. Prophylactic strategies are promising but should be described with their nonrandomized evidence and limitations. Multimodal surveillance linked to patient-specific dosimetry is more defensible than an OCTA-only algorithm.
